# Evaluation of the Diagnostic and Prognostic Value of Syndecan-1 in Acute Leukemia Patients

**DOI:** 10.7759/cureus.10594

**Published:** 2020-09-22

**Authors:** Reham Alghandour, Mohamed A Ebrahim, Hayam Ghazy, Sameh Shamaa, Ziad Emarah, Mohammed M Al-Gayyar

**Affiliations:** 1 Medical Oncology, Internal Medicine Department, Mansoura University Faculty of Medicine, Mansoura, EGY; 2 Medical Oncology Unit, Oncology Center, Mansoura University, Mansoura, EGY; 3 Department of Pharmaceutical Chemistry, University of Tabuk Faculty of Pharmacy, Tabuk, SAU; 4 Department of Biochemistry, Mansoura University Faculty of Pharmacy, Mansoura, EGY

**Keywords:** acute lymphoblastic leukemia (all), acute myeloid leukemia (aml), diagnosis, prognosis, syndecan-1 (cd138)

## Abstract

Syndecan-1 (also known as SDC-1 or CD138) is a transmembrane proteoglycan that is expressed in many hematological and solid tumors and affects the prognosis of those cancers. We conducted this study to investigate the prognostic role of syndecan-1 in acute leukemia. Forty cases of de novo acute leukemia patients, 24 with acute myeloid leukemia (AML) and 16 with acute lymphoblastic leukemia (ALL), presented at the Oncology Center of Mansoura University, Mansoura, Egypt, with a follow-up period of 26 months. Syndecan-1 was determined in serum and leukocytes by enzyme-linked immunosorbent assay (ELISA). The results from acute leukemia patients were compared with those of 15 healthy subjects. We observed that soluble syndecan-1 was higher in AML (median, 160.60 ng/ml) compared with ALL (median, 76.10 ng/ml) and healthy controls (median, 30.95 ng/ml). There was a significant correlation between syndecan-1 either in leukocytes or soluble form and response to treatment in patients with AML (p = 0.02 and p = 0.04, respectively), but these correlations were not statistically significant for ALL cases. Finally, there was a significant correlation between the soluble syndecan-1 level and overall survival in AML cases (p = 0.04), but the correlation was not significant for ALL cases. In conclusion, syndecan-1 is a useful biomarker for AML but not for ALL.

## Introduction

Acute leukemia is a heterogeneous hematological malignancy characterized by clonal proliferation of poorly differentiated and immature blast cells in bone marrow, blood, and/or other tissues. Acute leukemia is broadly classified as myeloid (AML) or lymphoblastic (ALL) leukemia [1]. AML is the most common form of acute leukemia among adults and represents the largest number of annual deaths from leukemia in the United States [2]. Acute leukemia is a class of diseases with a common aggressive clinical presentation but with prognosis and management that is dependent upon underlying genetic and molecular characteristics of the neoplasm [3]. Therefore, new molecular markers are needed to refine the prognosis.

Syndecan-1 (also known as SDC-1 or CD138) is a transmembrane proteoglycan that is normally present on the luminal surface of endothelial cells. Because it constitutes the backbone of the glycocalyx vascular barrier, it is responsible for cell-to-cell adhesion [4]. Increased levels of circulating syndecan-1 in the blood are correlated with glycocalyx degradation, which occurs in various conditions such as pregnancy, ischemia, sepsis, and inflammation [5].

Several studies examined the impact of syndecan-1 in various neoplasms and concluded that its altered expression often led to malignant neoplasms [6]. This was attributed to increased cell proliferation, growth, invasion, cell survival, metastasis, and angiogenesis [7,8]. Accumulating evidence suggests that syndecan-1 is involved in the stimulation of cancer stem cells or tumor-initiating cells, and this may affect disease relapse and resistance to chemotherapy [9]. Overexpression of syndecan-1 is reported in many hematological and solid tumors such as multiple myeloma, non-Hodgkin's lymphoma, and breast, bladder, pancreatic, lung, ovarian, endometrial, and hepatocellular carcinoma, and its expression may affect the prognosis of those cancers [6].

Inside the bone marrow, syndecan-1 is detected on the surface of the B lymphocyte cell lineage, and its expression changes at specific stages of differentiation [10]. Moreover, hematopoietic stem cells and leukemic stem cells express numerous adhesive molecules, including syndecan-1 [11]. The expression of syndecan-1 is restricted to plasma cells [12]. Deregulation of adhesion is considered a hallmark of metastatic solid tumors, but it is not observed in acute leukemia because acute leukemia is a heterogeneous group of diseases originating from multiple causes [11]. Notably, the alteration of expression and signaling of adhesive molecules and their ligands has a role in leukemia development and progression, and these may serve as possible potential sites for target therapy [11].

Interestingly, many ongoing clinical trials are exploring the safety and efficacy of targeting syndecan-1 in multiple myeloma [13,14]. Therefore, we measured the levels of syndecan-1 in AML and ALL by measuring syndecan-1 expression on leukocytes and its plasma level (soluble syndecan-1). We correlated the results with biochemical parameters for acute leukemia and leukemia prognosis.

## Materials and methods

Patients

Forty de novo acute leukemia patients were recruited from the Oncology Center at Mansoura University, Egypt, and followed up for 26 months. The study was approved by the local institutional ethical committee, and patient consent was obtained according to the regulations of the Egyptian Ministry of Health. The inclusion criteria of the study were any de novo patients diagnosed with acute leukemia (myeloid or lymphoid), that did not receive previous chemotherapy, with age ranging from 16 to 80 years. The exclusion criteria were any patients with chronic or hairy cell leukemia, patients associated with other types of malignancy, or those with severe organ dysfunction. The cases included in the study were divided into two groups, as follows. Group 1 consisted of 24 AML patients, 15 cases (62.5%) were male, and nine cases (37.5%) were female, and the median age was 50 years old. Group 2 consisted of 16 ALL patients, 12 cases (75%) were male, and four cases (25%) were female, and the median age was 25 years. The clinicopathological parameters of the studied cases in both groups are summarized in Tables 1 and 2. In addition, the control group consisted of 15 healthy individuals, consisting of nine females and seven males with the same age range of the patients included in the study, and no apparent evidence of active disease or medical disorders.

**Table 1:** Clinicopathologic features of studied cases AML: acute myeloid leukemia, ALL: acute lymphoblastic leukemia, BM: bone marrow, Hb: hemoglobin, WBC: white blood cells.

	AML		ALL	
	N	%	N	%
Gender				
Male	15	62.5%	12	75%
Female	9	37.5%	4	25%
Performance status				
0-2	16	66.7%	14	87.5%
3-4	8	33.3%	2	12.5%
Bleeding				
Absent	19	79.2%	9	56.3%
Present	5	20.8%	7	43.8%
Lymphadenopathy				
Absent	16	66.7%	9	56.3%
Present	8	33.3%	7	43.8%
Splenomegaly				
Absent	9	37.5%	5	31.3%
Present	15	62.5%	11	68.8%
Central nervous system involvement				
Absent	23	95.8%	13	81.3%
Present	1	4.2%	3	18.8%
BM cellularity				
Normal	7	41.2%	0	0%
Hyper	10	58.8%	10	100%
Clinical results	AML		ALL	
	Median	Range	Median	Range
Age	50	18-66	25	15-58
Hb conc. (g/dl)	6.8	3.8-13.5	8.2	5.2-13.9
WBCs x 1000/µL	8.4	8-229.0	34.3	2.1-129.0
Platelets x 1000/µL	40	8-335	42	7-297
BM blast (%)	70	23-95	78	31-95

**Table 2:** Level of syndecan-1 in studied cases and control group AML: acute myeloid leukemia, ALL: acute lymphoblastic leukemia.

	AML		ALL		Control		p-value
	Median	Range	Median	Range	Median	Range	
Leukocyte syndecan-1 (ng/ml)	30.35	18.35-163.35	29.85	17.35-82.85	23.85	15.15-42.85	0.1
Plasma syndecan-1 (ng/ml)	160.60	23.35-1481.35	76.10	22.85-695.35	30.95	21.85-455.85	<0.0001
Post hoc tests for plasma syndecan-1 (ng/ml)	AML vs. Control, p = 0.001; ALL vs. Control, p = 0.012; AML vs. ALL, p = 0.87						

Blood sampling

Fasting blood was collected by venipuncture from each patient and control subject. Samples were left to clot for 20 to 30 minutes at room temperature, followed by centrifugation at 1500 rpm for 10 minutes. Plasma was transferred to a polypropylene tube, frozen, and maintained at −80 °C until use. Leukocytes were separated and washed once with phosphate-buffered saline (PBS). The final leukocytes were suspended in PBS and were maintained at −20 °C for further investigations.

Enzyme-linked immunosorbent assay

The human serum and leukocyte syndecan-1 concentrations were determined by ELISA assay using a commercially available kit (Abcam plc., Cambridge, USA).

Statistical analysis

Descriptive statistics included mean ± standard deviation or median and range as appropriate for continuous variables. The Shapiro-Wilk normality test was applied to determine if the values of the continuous variables were normally distributed and obtain the frequency (%) for categorical variables. Student's t-test or Mann-Whitney U-test were used for comparison of continuous variables between groups, and the Kruskal-Wallis test was used to compare the distributions between more than two groups. Correlations between variables were determined by Kendall's tau non-parametric correlation coefficient, while categorical data were compared between groups by using the chi-square test and Fisher's exact or chi-square test with Yates correction. Pearson's or point-biserial correlation test was used to obtain correlations, as appropriate. The differences were considered significant at two-tailed p < 0.05. The probability of disease-free survival (DFS) and overall survival (OS) was defined with Kaplan-Meier's method, and differences were compared with the log-rank test. Multivariate analysis for DFS was carried out using the Cox proportional hazards model, in which some potential prognostic factors were included. Follow-up was defined from enrollment to the last follow-up. All statistical computations were performed using SPSS Statistics for Windows, Version 18.0 (SPSS, Inc., Chicago, USA).

## Results

Study patients

The clinicopathological features of the studied cases are summarized in Table 1. All patients with AML were treated with a standard 7+3 protocol for chemotherapy except two cases. In addition to the 7+3 protocol, one patient also received subcutaneous cytosine arabinoside (Ara C), and the other also received all-trans-retinoic acid. During treatment, 47.6% of patients underwent complete remission (CR), but unfortunately, 70% of them relapsed. At the end of the follow-up period, 16 patients died in this group.

Six patients with ALL were treated with an augmented Berlin-Frankfurt-Munster protocol, and 10 patients were treated with the hyperfractionated cyclophosphamide, vincristine, doxorubicin, and dexamethasone protocol alternating with high-dose methotrexate and Ara C. During treatment, 14 patients (87.5%) underwent CR, but unfortunately, seven patients relapsed. At the end of the follow-up period, eight patients (50%) died.

Cytogenetic studies on pretreatment of bone marrow or unstimulated blood samples were performed. Fluorescence in situ hybridization was used to detect favorable cytogenetics in AML and ALL patients. We found that for AML patients, t(8;21) was positive in two cases (8.3%), t(15;17) was positive in one case, and inv(16) was positive in one case. For ALL patients, we determined that t(9;22) was positive in two cases, for which imatinib was added to the treatment protocol.

Leukocytic expression and plasma level of syndecan-1

Expression of syndecan-1 on leukocytes was higher in AML than ALL patients, as syndecan-1 was expressed on leukocytes at a median of 30.35 ng/ml (range, 18.35-163.35 ng/ml) in AML cases, and 29.85 ng/ml (range, 17.35-82.85 ng/ml) in ALL cases. In addition, the plasma level of syndecan-1 (soluble form) in AML cases was higher than that in ALL cases. The median level of soluble syndecan-1 was 160.60 ng/ml (range, 23.35-1481.35 ng/ml) in AML, while it was 76.10 ng/ml (range, 22.85-695.35 ng/ml) in ALL cases. We noted that when we compared the level of soluble syndecan-1 among the three groups (AML, ALL, and control group), it was very significant (p < 0.0001). When we compared the expression of syndecan-1 on leukocytes among the three groups, it was not significant (p = 0.1). Moreover, the post hoc test indicated that there were high levels of soluble syndecan-1 in AML patients compared to the control group with a very significant difference (p = 0.001). In addition, ALL patients had a high-soluble syndecan-1 level compared to the control group with significance (p = 0.012). However, there was no significant difference in the plasma level of syndecan-1 in AML versus ALL cases (p = 0.87; Table 2).

Correlation of syndecan-1 level with clinicopathological parameters in acute leukemia patients

AML cases with high expression of syndecan-1 on leukocytes were older (r = 0.41, p = 0.048) and associated with high leukocytic counts (r = 0.46, p = 0.024). However, there was no significant correlation between the expression of syndecan-1 on leukocytes and platelet count, bone marrow (BM) blasts, or hemoglobin (Hb) concentration (Table 3). There was no significant correlation between the soluble level of syndecan-1 and age, leukocytic count, Hb concentration, platelet count, or BM blasts in AML patients. In parallel, we found that there was no significant correlation between the level of expression of syndecan-1 on leukocytes or its soluble level and age, leukocytic count, BM blast, Hb concentration, and platelet count in ALL patients (Table 3).

**Table 3:** Correlation between syndecan-1 and study parameters in acute leukemia cases AML: acute myeloid leukemia, ALL: acute lymphoblastic leukemia, BM: bone marrow, Hb: hemoglobin, WBC: white blood cells.

		Plasma syndecan-1 (ng/ml)	Age	WBCs × 1000/µL	Hb conc. (g/dl)	Platelets × 1000/µL	BM blast %
AML							
Leukocyte syndecan-1 (ng/ml)	r	0.43	0.41	0.46	0.36	0.176	0.208
	p	0.037	0.048	0.024	0.08	0.411	0.330
Plasma syndecan-1 (ng/ml)	r	1.000	0.13	0.250	-0.088	-0.314	0.188
	P	0.0	0.51	0.238	0.682	0.135	0.380
ALL							
Leukocyte syndecan-1 (ng/ml)	r	-0.418	0.389	-0.217	-0.211	0.090	0.387
	p	0.107	0.137	0.419	0.432	0.740	0.139
Plasma syndecan-1 (ng/ml)	r		-0.260	0.161	0.261	0.127	-0.317
	p		0.332	0.552	0.329	0.639	0.232

The relationship between syndecan-1 level and response to treatment in acute leukemia patients

**Figure 1:**
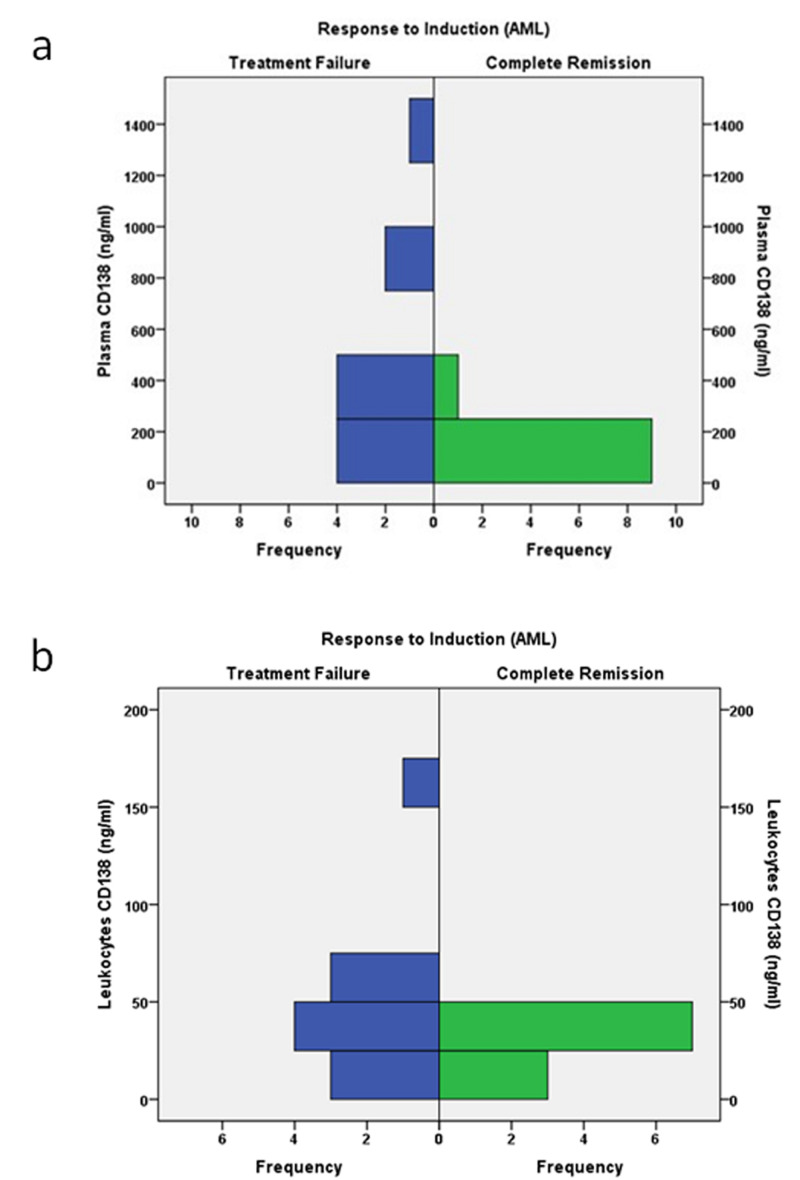
Relationship of (a) plasma syndecan-1 (CD138) and (b) CD138 leukocytes in response to induction chemotherapy in AML cases AML: acute myeloid leukemia.

In AML cases, there was a significant relationship between the expression of syndecan-1 on leukocytes and the response to treatment, with either failure or achievement of CR (p = 0.02). In addition, there was a significant relationship between the soluble level of syndecan-1 and the response to treatment, with either failure or achievement of CR (p = 0.04). However, in ALL patients, there was no significant relationship between the soluble level of syndecan-1 or its expression on leukocytes and the response to treatment (p = 1, p = 0.33; Table 4 and Figure 1).

**Table 4:** Relationship between syndecan-1 and response to induction chemotherapy in acute leukemia cases AML: acute myeloid leukemia, ALL: acute lymphoblastic leukemia.

	Treatment failure		Complete remission		p-value
	Median	Range	Median	Range	
AML					
Leukocyte syndecan-1 (ng/ml)	37.85	23.85-163.35	28.10	18.35-35.35	0.02
Plasma syndecan-1 (ng/ml)	355.35	28.35-1481.35	110.10	23.35-296.85	0.043
ALL					
Leukocyte syndecan-1 (ng/ml)	55.10	27.35-82.85	29.60	17.35-62.35	0.33
Plasma syndecan-1 (ng/ml)	219.35	22.85-415.85	76.10	37.85-695.35	1.0

The relationship between syndecan-1 level and assessment of survival rates in studied patients

**Figure 2:**
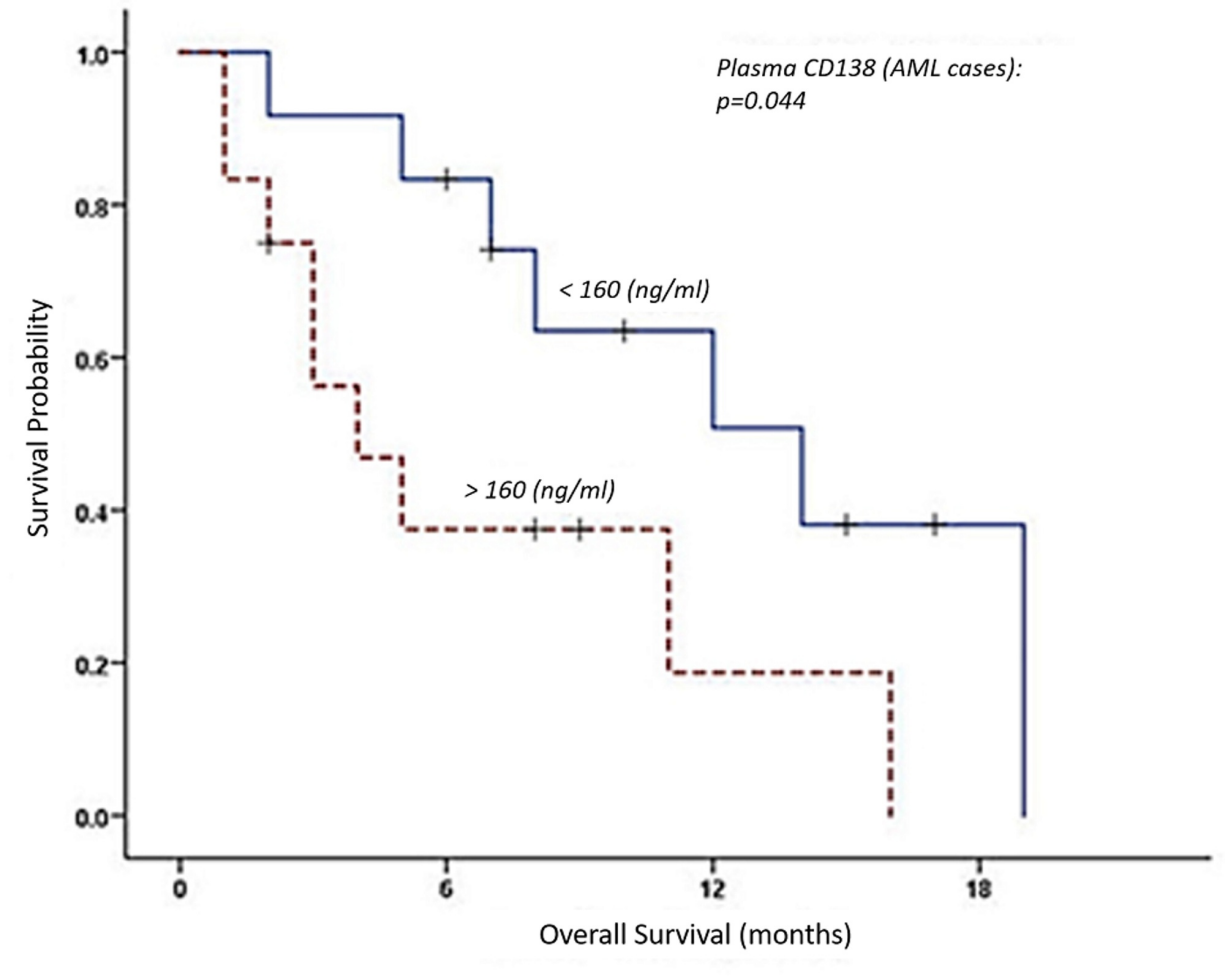
Kaplan-Meier curve showing overall survival of studied AML cases stratified according to plasma CD 138 level (above or below the median cut off) AML: acute myeloid leukemia.

At the end of the follow-up period (26 months), the median DFS of studied AML and ALL cases was six months (95% CI, 3.2-8.7 months) and 14 months (95% CI, 10.1-17.9 months), respectively. The median OS of studied AML and ALL cases was 11 months (95% CI, 3.87-18.13 months) and 14 months (95% CI, 5.34.1-22.65 months), respectively. We divided AML and ALL patients into two groups according to their level of syndecan-1. The median cut-off plasma level was 160.60 ng/ml in AML cases and 76.10 ng/ml in ALL cases, while the median cut off level of expression of syndecan-1 on leukocytes was 30.35 ng/ml in AML cases and 29.85 ng/ml in ALL cases. We observed that there was no significant relationship between median DFS and syndecan-1 level when comparing either its plasma level or its expression on leukocytes in AML and ALL cases. However, when we assessed the relationship of OS and syndecan-1 level, there was a significant difference in the median OS of studied AML cases when we compared the OS at low plasma level of syndecan-1 (14 months) and high plasma level (four months; p = 0.044; Figure 2). However, there was no significant relationship between median OS and expression of syndecan-1 on leukocytes in AML cases. Also, in ALL, there was no significant relationship between median OS and syndecan-1 level, when comparing either its plasma level or its expression on leukocytes.

## Discussion

Syndecan-1 was highly expressed in leukocytes and released in plasma of AML and ALL patients [15,16]. A high level of circulating syndecan-1 in AML patients, which was observed in our study, was in agreement with a previous study [17]. However, to the best of our knowledge, assessment of the serum level of syndecan-1 in ALL patients has not been previously performed. We observed that on leukocytes, the level of syndecan-1, comprising either soluble syndecan-1 or its expression, was higher in AML than that in ALL. Also, AML patients exhibited higher expression of syndecan-1 on leukocytes or high plasma or circulating levels of syndecan-1 (r = 0.43, p = 0.037). However, in ALL patients, there was no significant correlation between the expression of syndecan-1 on leukocytes and its plasma level. This observation could be explained by the alteration of the level of heparanase and other proteases that are responsible for the shedding of syndecan-1 from the cell surface to the serum [18,19].

Interestingly, in AML cases in the current study, there was a significant relationship between the expression of syndecan-1 on leukocytes and the response to treatment, consisting of either failure or achievement of CR (p = 0.02). Also, there was a significant relationship between the soluble level of syndecan-1 in the studied AML cases and the response to treatment, with either failure or achievement of CR (p = 0.04). Thus, we concluded that AML patients who overexpressed syndecan-1 on leukocytes or had a high plasma level of syndecan-1 failed to achieve CR. However, there was no significant correlation in ALL cases between the level of syndecan-1 and the response to treatment. This observation might be explained by the tumor exhibiting higher expression of syndecan-1, and thus, was more resistant to chemotherapy followed by subsequent treatment failure [20]. Ramani and Sanderson observed that myeloma patients with higher expression of syndecan-1 on the cell surface were chemoresistant [20]. The resistance might have occurred because the chemotherapy used in the treatment of myeloma stimulated the synthesis and shedding of syndecan-1, which led to the accumulation of high levels of syndecan-1, thus enhancing relapse and promoting tumor progression. The authors also reported this observation in pancreatic cancer cells as well, indicating that drug-induced shedding of syndecan-1 might occur in many cancer types [20]. Interestingly, doxorubicin, which is also used in the treatment of acute leukemia, is one of the chemotherapeutic agents that enhance the shedding of syndecan-1 [21].

In contrast to our results, Larsen et al. found that syndecan-1 was not predictive of the outcome in AML patients, but it has more clinical significance because a high expression of serum syndecan-1 is associated with bleeding, thrombocytopenia, leukocytosis, and endothelial cell damage [17]. We noted in our study that AML patients exhibited high levels of syndecan-1 compared to the control group (p = 0.001). ALL patients also had a high syndecan-1 level compared to the control group (p = 0.012), which indicates that syndecan-1 has the potential to be a useful biomarker for acute leukemia. However, it cannot be used as a diagnostic marker that differentiates AML from ALL because there was no significant value when measured in AML versus ALL (p = 0.87). Kim et al. showed that the use of soluble syndecan-1 as a tool for diagnosing myeloma has limitations because soluble syndecan-1 was not elevated in all myeloma patients. However, the authors indicated that the baseline levels of syndecan-1 did not predict therapeutic response in those patients [22].

In the current study, at the end of the follow-up period with median cut-off plasma levels of syndecan-1 of 160 ng/ml in AML cases, there was a significant difference in the median OS of patients with low plasma levels of syndecan-1 (14 months) and high plasma levels (four months; p = 0.044). We found that there was no significant difference in the median OS of studied ALL cases when we compared the OS in patients with low and high plasma levels of syndecan-1 (p = 0.26).

Seidel et al. identified soluble syndecan-1 as an independent prognostic marker in a series of 174 myeloma patients [23]. In their study, they divided the patients into two groups according to the level of soluble syndecan-1, with a median cut-off level of syndecan-1 of 299.52 ng/ml. The high syndecan-1 level group had a median survival of 20 months, while the low-level group had a median survival of 44 months (p < 0.0001) [23]. Additionally, Kumar et al. reported significant differences in the progression and overall progression-free survival in a larger study of more than 501 myeloma patients with a median cut-off level of syndecan-1 of 158 ng/ml. The high syndecan-1 group exhibited a median survival of 36.3 months compared with 49.3 months for the low syndecan-1 level group (p < 0.0001) [24]. Furthermore, in mice, increased expression of syndecan-1 is associated with a therapy-resistant blastic crisis clone of chronic myeloid leukemia, and its loss is associated with marked improvement in survival [25].

## Conclusions

Syndecan-1 was significantly elevated in AML either in leukocytes or soluble form and it was correlated with overall survival of AML patients. Therefore, it a useful prognostic marker in AML. Although the levels of syndecan-1 are elevated in ALL patients, there is no significant correlation between the leukocytes and serum levels of syndecan-1 in ALL patients. In addition, there was no significant correlation between syndecan-1 levels and the survival of ALL patients. Finally, we observed that the level of expression of syndecan-1 on leukocytes and its plasma level were more prominent in AML than in ALL cases. Therefore, syndecan-1 has limitations in differentiating between both types of acute leukemia.
